# Editorial: Nurturing sustainable nutrition through innovations in food science and technology

**DOI:** 10.3389/fnut.2022.999055

**Published:** 2022-08-09

**Authors:** Giuseppe Poli, Mélanie Charron, Carlo Agostoni, I. Sam Saguy

**Affiliations:** ^1^Unit of General Pathology and Physiopathology, Department of Clinical and Biological Sciences, San Luigi Hospital, University of Turin, Turin, Italy; ^2^Soremartec Ferrero Group, Alba, Italy; ^3^Pediatric Intermediate Care Unit, IRCCS Ca' Granda, Ospedale Maggiore Policlinico, Milan, Italy; ^4^Department of Clinical Sciences and Community Health, University of Milan, Milan, Italy; ^5^Robert H. Smith Faculty of Agriculture, Food and Environment, The Hebrew University of Jerusalem, Jerusalem, Israel

**Keywords:** nutrition and sustainability, innovation, palm oil, milk, oxysterols, ultra-processed foods, sensory, microbiome

## Introduction

Nutrition Science and Food Science and Technology (FS&T) are at the heart of disruptive evolutionary processes, and exponential progresses in science, health, innovation and digital technology. Suitable knowledge of these advances is still evolving. Hence, a paradigm shift and novel curricula are required. Such an attempt was made in the 2020/21 International Master Michele Ferrero 8th edition program offered by the Ferrero Foundation and Soremartec in collaboration with the University of Turin and the Catholic University of the Sacred Heart, Milan. A series of nine-webinars by more than 30 invited eminent scientists and key industrial leaders was tailored-made, offering a wide spectrum of the state of the art knowledge and views.

This Research Topic directly stems from the 8th Master edition, with a Research Topic of papers comprehensively covering all FS&T-related key and novel topics elaborated during the Course. In light of the plethora of reviews published on the selected topics, challenging to read and digest by many practitioners, special efforts have been placed on offering concise yet comprehensive contributions.

a. Position paper

“*A need for a paradigm shift in healthy nutrition research*” by Aleta et al..

Research in sustainable and healthy nutrition requires the application of the latest advances in seemingly unrelated domains, such as complex systems and network sciences on one hand and big data and artificial intelligence on the other. Focusing here primarily on nutrition and health, the methodological changes needed to open current disciplinary boundaries to the methods, languages, and knowledge of the digital age are discussed, laying the groundwork for the development of a systems thinking approach. Specifically, a paradigm shift is required toward adoption of interdisciplinary, complex-systems-based research to tackle the immense challenges required for dealing with an evolving interdependent multiple scale systems. The latter are ranging from the metabolome to the population level, heterogeneous and more often than not contained incomplete data. Also, population changes subject to many behavioral and environmental pressures. To illustrate the importance of this methodological innovation, the paper focuses on the consumption aspects of nutrition rather than production. Nevertheless, the importance of system-wide studies that involve both these components of nutrition are recognized. Specific research directions that would make it possible to find new correlations and, possibly, causal relationships across scales, and in addition, answering pressing questions in the area of sustainable and healthy nutrition are furnished.

b. Perspective articles

“*Plant-based: A perspective on nutritional and technological issues. Are we ready for “precision processing?”*” by Menta et al..

Nutrition science is facing challenging times due to accelerated changes and fast-growing global population in the coming years. Innovation in raw materials, processes, science and digital capabilities seems to be the key to address this new era, and, in this context, a plant-based approach to nutrition could meet the needs of these new challenges. Particular attention is focused on highlighting the differences in quality and functionality of animal and plant proteins, along with a call to unite global efforts and to offer suitable available solutions.

“*Food products and digital tools: The unexpected interconnections*” by Marra.

The current advances and future directions in the use of science-based digital tools in food product design are highlighted, first, an overview of studies exploring food related apps and social media for understanding consumers' perception and preferences, and, second, a discussion on the integration of the derived data. A wider scheme for food product design based on predictive features is needed, using advanced multiscale and hybrid methods. Linking product features with the understanding of consumers' needs and preferences offers significant benefits for start-uppers and researchers who develop tools for reinventing food product design.

c. Mini reviews with a particular focus on*:*

- Health and consumers behavior impact

“*Impact of dietary palmitic acid on lipid metabolism*” by Murru et al..

Palmitic acid (PA) is ubiquitously present in dietary fat guaranteeing the relative high requirement and content in the human body, with a crucial physiological role. Lower placental transfer of PA strongly induces its endogenous biosynthesis from glucose *via de novo* lipogenesis (DNL), securing a tight homeostatic control of PA tissue concentrations. Unbalanced body composition and reduced physical fitness might be either cause or consequence of DNL. Unbalanced saturated fats/ polyunsaturated fatty acids intake may further impact the energetic and metabolic balance.

“*Dietary fats, human nutrition, and the environment: Balance and sustainability*” by Meijaard et al..

Optimization of oil or fat choices that most benefit health and the environments in areas where these are produced, is undermined by a significant lack of data. The article reviews current knowledge about the sustainability impacts of oils and fats, focusing the role of biodiversity in their production. Gaps in knowledge and analytical to address them are highlighted.

“*Oxysterols as reliable markers of quality and safety in cholesterol containing food ingredients and products*” by Canzoneri et al..

Cholesterol is a lipid of functional value that easily undergoes oxidation, leading to a wide variety of cholesterol oxidation products, named oxysterols. Recent research points to oxysterols as highly reliable markers of food quality, before/after industrial processes and storage. Survey of relevant literature highlighted the advantages of quantifying oxysterols as quality markers of food and food ingredients. Further, bioavailability, metabolism, and pathophysiological features of measured oxysterols are fully discussed.

“*A2 milk and BCM-7 peptide as emerging parameters of milk quality*” by Giribaldi et al..

Due to natural genetic variation, beta-casein (up to ~30% of the total protein) can be present in cows' milk as two distinct forms, A1 or A2 that differ for a single but crucial amino acid substitution. Only in A1 or A1A2 beta-casein containing milk and dairy products, the peptide β-casomorphin-7 (BCM-7) is released upon intestinal digestion. Such BCM-7 release has been associated with inflammatory disturbances of the gastrointestinal tract with alteration of the gut microbiota. The health ramifications of milk containing A2 β-casein subtype are highlighted.

“*Evolution of milk consumption and its psychological determinants*” by Castellini and Graffigna.

The food industry has developed new products in order to respond to consumer's needs and expectations, in spite of Institutional recommendations. The widespread consumption of lactose-free products (LFP) is therefore unjustified considering either the scientific evidence and the consumers' perspective. The review highlights the research findings related to the motives and psychological factors mainly influencing consumers to prefer LFP.

“*Milk: A scientific model for diet and health research in the twenty-first century*” by German et al..

Milk's glycans highlight the Darwinian pressure on lactation as a complete, nourishing and protective diet. These polysaccharides reach undigested the lower intestine where bacteria compete to release the monosaccharides and ferment them. *B. infantis*, is uniquely equipped with a repertoire of genes encoding enzymes capable of metabolizing the complex glycans of human milk. The intestinal microbiome dominated by *B. infantis*, shields the infant from the growth of enteropathogens and their endotoxins as a clear health benefit. The paper highlights how scientists should guide the future of agriculture and food in response to twenty-first century challenges to produce a food supply at the same time nourishing, safe and sustainable. Lactation provides an inspiring model of what future research strategy could be.

- Sensory cues and taste

“*Influence of sensory properties in moderating eating behaviors and food intake*” by Forde and de Graaf.

An overview of recent findings and opportunities to use foods' sensory properties as a “functional” component that may help promoting healthier eating habits while maintaining eating pleasure. Addressing the serious public health challenges posed by the modern food environment will require changes in food formulation and intake behavior. The utilization of foods sensory properties may support healthier food choices and intakes while suggesting a better management of chronic conditions such as obesity and type-2 diabetes.

“*Extra-oral taste receptors: function, disease, and perspectives*” by Behrens and Lang.

A brief introduction into human taste perception, receptive molecules and signal transduction is highlighted. The five basic taste qualities (i.e., salty, sour, sweet, umami, and bitter) provide important information on the energy content, the concentration of electrolytes and the presence of potentially harmful components in food items. Taste receptors in the gastrointestinal tract, participate in a variety of bioprocesses with meaningful effects on health too. Accordingly, complex selective forces may have contributed to shape taste receptors during evolution.

- Microbiome studies

“*Diet and gut microbiome and the “chicken or egg” problem*” by Daniel.

A concise and provocative scientific argument for a more comprehensive assessment of the individual's intestinal phenotype in microbiome studies to resolve the “chicken or egg” problem that has emerged from observational studies on functional effects is provided. It highlights that quantity and quality of the intestinal and fecal microbiome vary considerably between individuals and are dependent on a wide spectrum of intrinsic and/or environmental factors.

### Controversial aspects of the modern food era: Processes, dietary impact, and nutritional recommendations

“*Food innovation in the frame of circular economy by designing ultra-processed foods optimized for sustainable nutrition*” by Capozzi.

Circular economy emerges as a crucial driving force considering the future predicted world population growth. It highlights the necessity for finding the resources essential to produce food in sufficient quantity and quality, looking for sustainable sources, while trying to exploit all the value obtainable from raw materials. Nutritional studies will stimulate the selection of sources richer in nutrients and bioactive molecules. The assessment of the quality and safety of functional foods based on ingredients derived from food waste requires a more robust validation by means of the food-omics approach, which considers not only the composition of the final products but also the structural characterization of the matrix, as the bioaccessibility and the bioavailability of nutrients are strictly dependent on the functional characteristics of the innovative ingredients. This new approach offers a new avenue to assess relationships between circular economy and UPFs. Suitable solutions are proposed, analyzed, and discussed.

“*Integrating dietary impacts in food life cycle assessment*” by Jolliet.

Food production and food consumption have been too long studied separately. The review highlights how nutrition affects human health and states that environmental impacts of the entire food production and consumption can and should be consistently and systematically assessed, on a life cycle-based and a health-based perspective. The review also provides a novel approach to calculate the Health Nutrient Index score expressed in minutes (gained/lost) of healthy life per serving. This integration combined with utilization of Big Data and machine learning methods will help reporting interactions among healthy and sustainable foods.

“*Dietary patterns vs. dietary recommendations*” by De Cosm et al..

The classic views of nutrient-based healthy recommendations should today be replaced by the holistic view of “whole food form” patterns, epidemiologically connected to indices of human health. The Mediterranean, New Nordic and Japanese diets, respectively, offer three possible paradigms of this novel approach.

The program of the Master Degree in “Innovation in Food Science and Technology—Michele Ferrero” focused on innovation, as a key characteristic of food system, sustainable nutrition, and personalized health and nutrition. A multidisciplinary approach and international perspective characterized the teaching activities. This unique collaboration between Academia and Industry should serve as a model highlighting a new mindset. The “old normal” pre COVID-19 has been disrupted, while embracing the “new normal” becoming the only way forward. Facilitating new mindset and leading future innovation processes, strategic considerations, and developing new partnerships are paramount. Program like the one offered by the Ferrero Foundation is paramount and a significant bridge and a platform in pursuing future challenges.

## Author contributions

IS, GP, and CA: conceptualization. IS and GP: writing—original draft preparation. IS, GP, CA, and MC: writing—review and editing. All authors contributed to the article and approved the submitted version.

## Conflict of interest

The guest editors declare that this Research Topic received limited partial founding from Soremartec Italia Srl. GP, IS, and CA were involved in Master Michele Ferrero academic course and webinars and received limited partial funding from Fondazione Piera, Pietro e Giovanni Ferrero and Soremartec Italia S.r.l. MC is an employee of Soremartec Italia S.r.l.

## Publisher's note

All claims expressed in this article are solely those of the authors and do not necessarily represent those of their affiliated organizations, or those of the publisher, the editors and the reviewers. Any product that may be evaluated in this article, or claim that may be made by its manufacturer, is not guaranteed or endorsed by the publisher.

